# A Case Report of a Child with Bell’s Palsy

**DOI:** 10.7759/cureus.2408

**Published:** 2018-04-02

**Authors:** Kamleshun Ramphul, Stephanie G Mejias, Yogeshwaree Ramphul-Sicharam, Ezatullah Hamid, Ruhi Sonaye

**Affiliations:** 1 Department of Pediatrics, Shanghai Xin Hua Hospital Affiliated to Shanghai Jiao Tong University School of Medicine, Shanghai, CHN; 2 Department of Pediatrics, Robert Reid Cabral Children's Hospital Affiliated to the University Iberoamericana Unibe School of Medicine; 3 Sir Seewoosagur Ramgoolam National Hospital; 4 Pediatrics, Al-Hayat; 5 Medical Doctor, Bharati Vidyapeeth Deemed University Medical College and Hospital , Sangli, India, Mumbai, IND

**Keywords:** pediatrics, bell's palsy, facial nerve

## Abstract

Bell’s palsy is a neuropathy involving the seventh cranial nerve, also known as the facial nerve. It is usually caused by traumatic, infective, inflammatory or compressive conditions on the nerve. Many cases are also with no identifiable etiologies and are classified as idiopathic. Acute inflammation and edema of the cranial nerve seven can lead to the compression and eventual ischemia. The most common viral cause of Bell’s palsy is herpes simplex virus but there are several reports of other viruses such as Epstein-Barr virus, human immunodeficiency virus and the hepatitis B virus involved in with similar presentation. Presentation of Bell’s palsy in the pediatric population is quite rare and this makes early recognition and proper treatment important. We present a case of a three-year-old male with Bell’s palsy.

## Introduction

Bell’s palsy is a neuropathy involving the seventh cranial nerve, also known as the facial nerve. It was first described by Dr. Charles Bell in 1821 [1]. It is usually caused by traumatic, infective, inflammatory or compressive conditions on the nerve. Many cases with no identifiable etiologies exist and are classified as idiopathic. Bell’s palsy is usually unilateral and can be complete or partial. Each side can be affected equally [2]. Acute inflammation and edema of the cranial nerve seven can lead to the compression and eventual ischemia. The most common site is in the labyrinth segment [3]. The most common viral cause of Bell’s palsy is herpes simplex virus (HSV) but there are several reports of other viruses such as Epstein-Barr virus [4], human immunodeficiency virus [5] and the hepatitis B virus [6] involved in with similar presentation. Presentation of Bell’s palsy in the pediatric population is quite rare and this makes early recognition and proper treatment important. We present a case of a three-year-old male with Bell’s palsy.

## Case presentation

**Figure 1:**
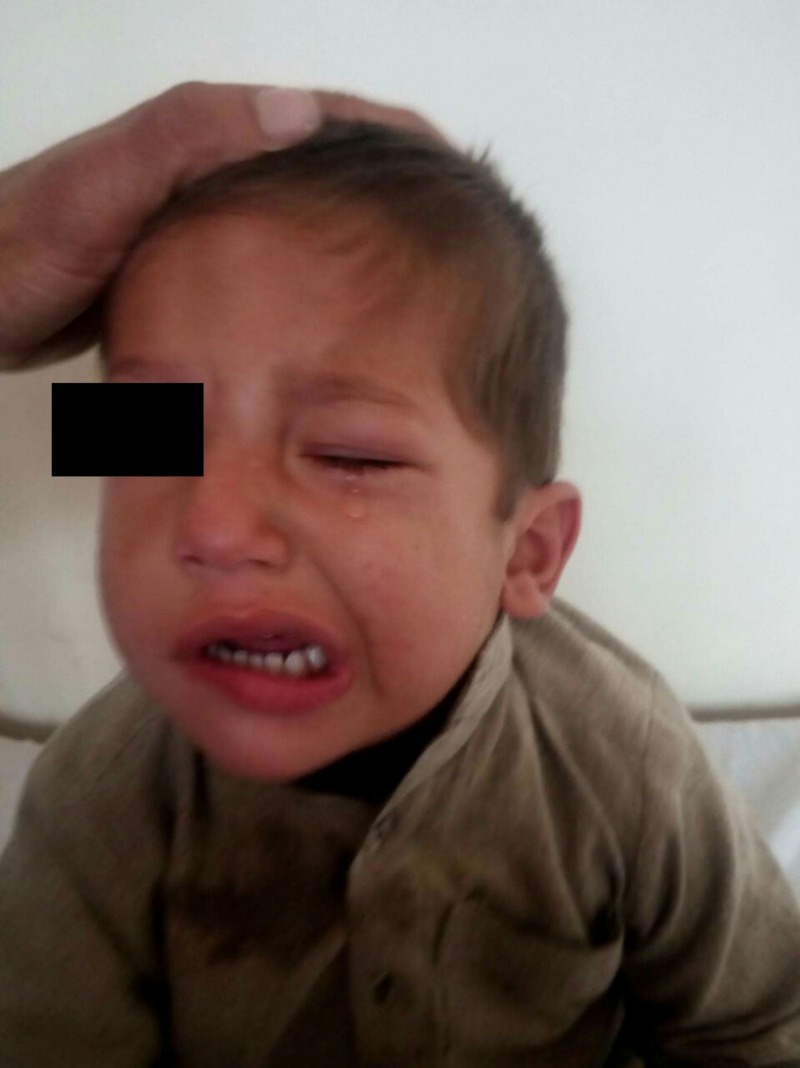
Three-year-old male presenting with left hemifacial Bell’s palsy.

A three-year-old boy, born by normal vaginal delivery from a nonconsanguineous union, presented to the pediatric centre in Afghanistan with left hemifacial palsy over the past month. His mother also reported that he had a swelling in his neck before the onset of the symptoms that resolved on its own. She denied any type of facial trauma or exposure to any sick contacts. The patient also did not report any recent fever, nausea, vomiting or other systemic symptoms. On physical examination, restricted movement of the left upper and lower lips was observed. Facial asymmetry with complete lower motor neuron type left facial nerve paralysis was observed involving the left eyes and eyebrows as seen in Figure 1. He denied any tenderness around that region. No active HSV or herpes zoster lesions were found in the oral mucosa. No lymphadenopathy was observed. Otoscopic examination revealed normal structures with no signs of infection. Further imaging of the face and neck also showed no marked clinically significant abnormalities. A diagnosis of left hemifacial Bell’s palsy of viral origin was made and he was started on a course of acyclovir, vitamin B complex, prednisolone and artificial tears. He was scheduled for a follow-up after six weeks and significant recovery was seen. Further follow-ups were planned for every six weeks for a year.

## Discussion

This patient’s response to acyclovir confirms the diagnosis of viral Bell’s palsy. There are multiple pathologies such as Ramsay Hunt Syndrome, tumors, Lyme disease and Sarcoidosis that were included in the differential diagnosis and ruled out eventually. Since most physicians rely on the clinical signs and symptoms for the diagnosis, proper exclusion of other possible causes should be done. Lyme disease usually presents with flu-like symptoms, “bulls-eye” rash of Erythema migrans and a Lyme antibody titre can rule out the disease. More invasive approaches such as lumbar puncture are usually avoided but should be considered for severe cases. A magnetic resonance imaging (MRI) or computed tomography (CT) can help locate any tumor. No IgG and IgM antibody titres were available at the centre to confirm the diagnosis of Bell’s palsy in our patient. The treating physician relied more on the clinical signs and symptoms to make his diagnosis and treat the patient. Proper follow-up also ensured that the child was properly monitored and the parents were advised to rush the child to the hospital if there were any signs and symptoms of his condition worsening.

Acyclovir is an antiviral that helped treat the cause of the Bell’s palsy in our patient. He was also administered prednisolone which helped decrease the inflammation [7] and artificial tears to keep the eyes moist and prevent keratitis. We also advised the parents to avoid exposure to any toxic fumes or dust during his recovery phase. While this patient recovered, physicians should also educate parents of warning signs. There are several documented cases where patients have lasting facial weakness and those with persistent clinical signs without improvement should undergo further investigation. While there are many conflicting opinions over the use of prednisolone along with acyclovir [8], the patient showed a good response to the combined therapy. Since children are vulnerable to the side effects of prednisolone, the dose should be small and properly tapered off [9].

## Conclusions

We report a case of Bell’s palsy seen in a three-year-old male who responded well with a combined therapy of prednisolone and acyclovir. Diagnosis was made mostly based on clinical findings and proper tests were carried out to rule out the possible differential diagnosis of the symptoms. Proper follow-ups were scheduled to monitor the progress of the patient. While the prognosis of this condition is usually good, parents should still be advised to look out for any deterioration in the signs and symptoms.
